# Supplementary figures and images for: Economic impact and disease burden of COVID-19 in a tertiary care hospital: A three-year analysis
Source: PLoS One. 2025 May 13;20(5):e0323200. doi: 10.1371/journal.pone.0323200 (PMC12074262; doi:10.1371/journal.pone.0323200)

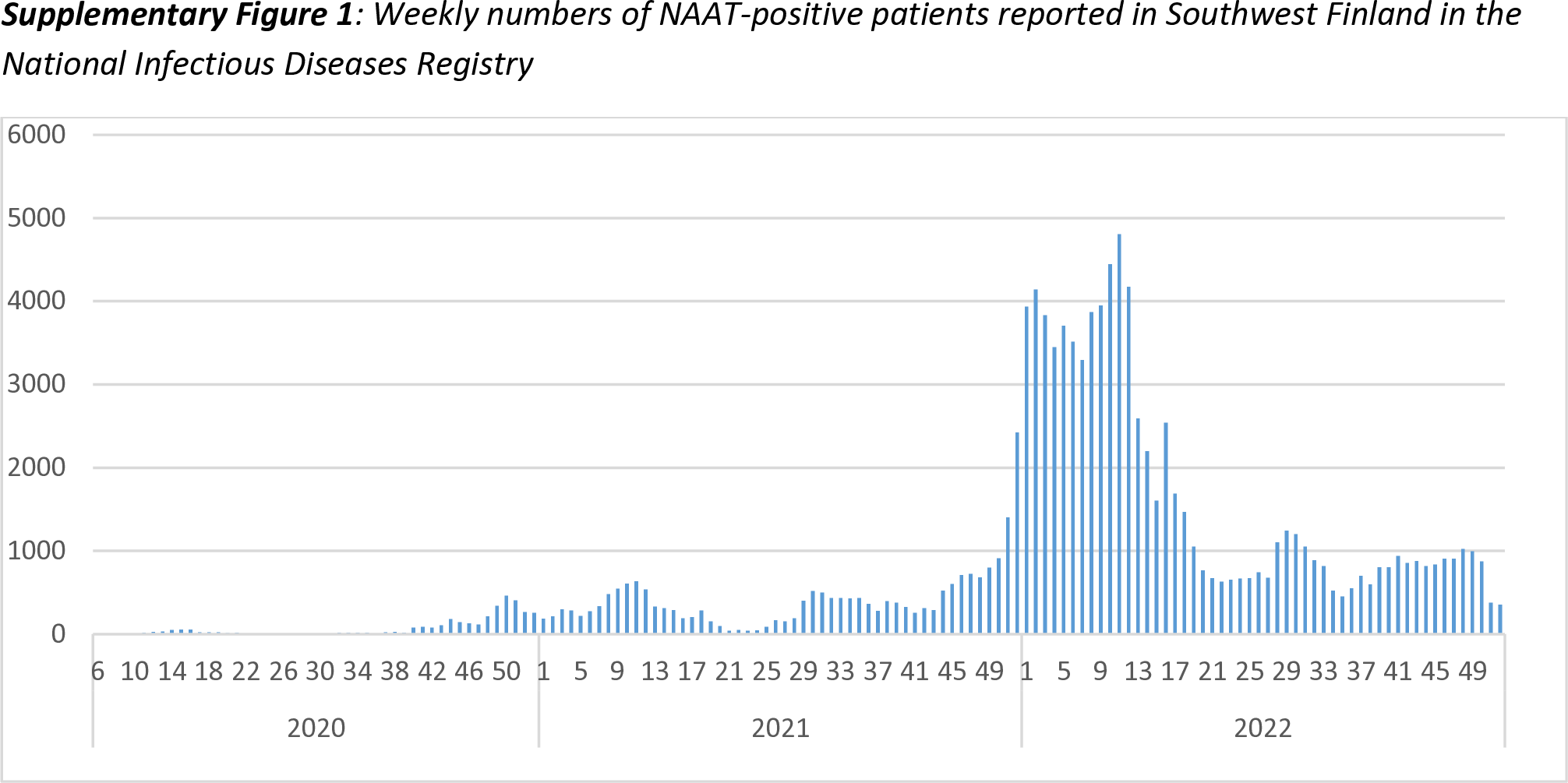

Supplement: S1 Fig — (TIF) [file pone.0323200.s001.tif]

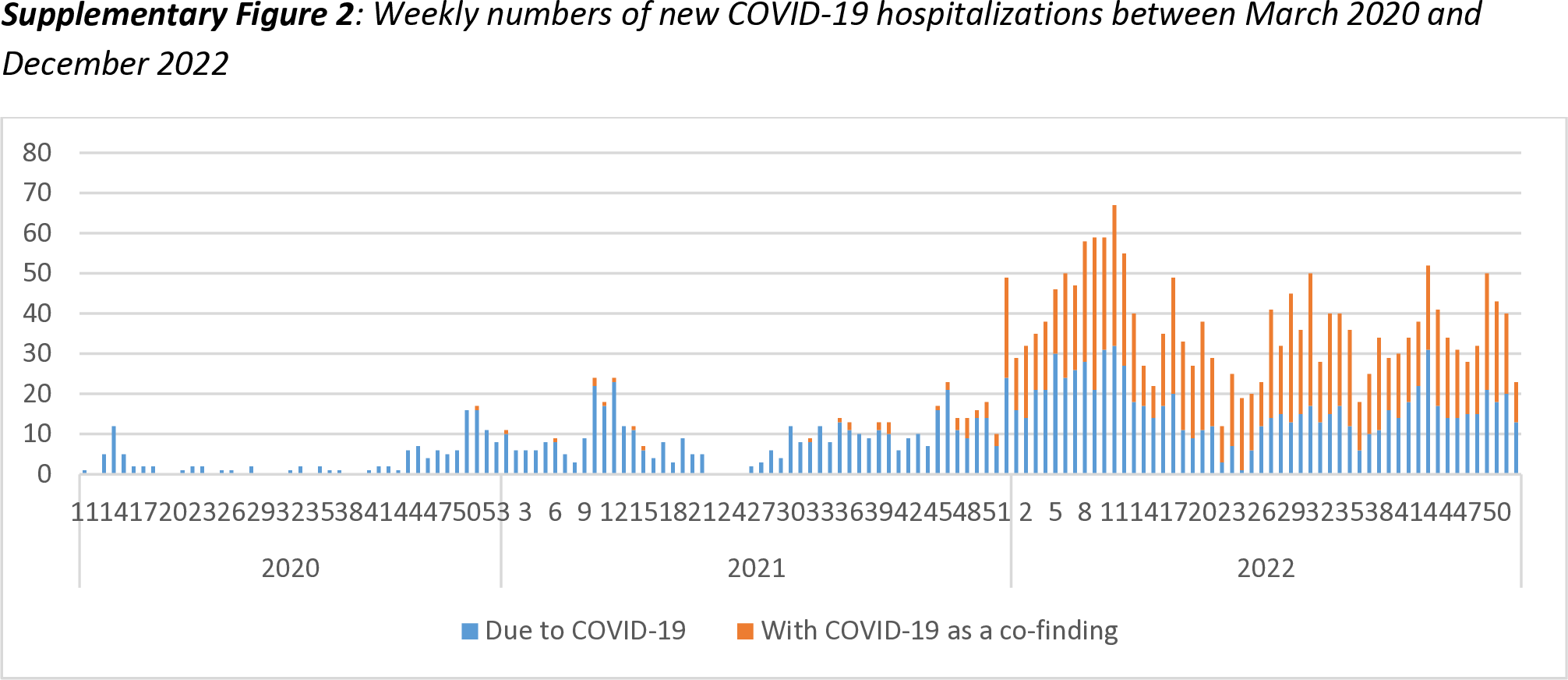

Supplement: S2 Fig — (TIF) [file pone.0323200.s002.tif]
