# Supplementary material for: Economic impact and disease burden of COVID-19 in a tertiary care hospital: A three-year analysis
Source: PLoS One. 2025 May 13;20(5):e0323200. doi: 10.1371/journal.pone.0323200 (PMC12074262; doi:10.1371/journal.pone.0323200)
Supplement: S1 Table — (DOCX) [file pone.0323200.s003.docx]

***Supplementary Table 1.*** *Annual characteristics of the COVID-19 pandemic and its control measures in Finland during 2020-2022*

|  | 2020 | 2021 | 2022 |
| --- | --- | --- | --- |
| **Prevailing virus variant** | Wild type virus | Alpha and delta variants | Omicron variants |
| **COVID testing coverage** | Good COVID-19 NAAT testing availability and coverage since summer | COVID-19 NAAT testing coverage good | Home COVID-19 antigen testing available since summer, community surveillance data incomplete |
| **Vaccine** | No vaccine protection | Vaccine program ongoing | 90% of adult population vaccinated, break through infections common |
| **Disease characteristics** | Mild to severe pulmonary symptoms | Mild to severe pulmonary symptoms | Severe pulmonary symptoms infrequent |
| **Control measures** | Isolation, community lock down | Isolation, community lock down | Less or nonexisting restrictions |
