# Supplementary material for: Economic impact and disease burden of COVID-19 in a tertiary care hospital: A three-year analysis
Source: PLoS One. 2025 May 13;20(5):e0323200. doi: 10.1371/journal.pone.0323200 (PMC12074262; doi:10.1371/journal.pone.0323200)
Supplement: S2 Table — (DOCX) [file pone.0323200.s004.docx]

***Supplementary Table 2****. Yearly numbers of patients hospitalized due to COVID-19 and with COVID-19 and the number and proportion of unvaccinated patients*

| Year of diagnosis | Due to COVID-19 | No (%) of patients unvaccinated | With COVID-19 | No (%) of cases unvaccinated | Total |
| --- | --- | --- | --- | --- | --- |
| 2020 | 132 | 132 (100%) | 11 | 11 (100%) | 143 |
| 2021 | 456 | 347 (76%) | 46 | 24 (53%) | 502 |
| 2022 | 856 | 274 (32%) | 1054 | 179 (17%) | 1910 |
| Total | 1444 | 753 (52%) | 1106 | 209 (19%) | 2555 |
