# Supplementary material for: Economic impact and disease burden of COVID-19 in a tertiary care hospital: A three-year analysis
Source: PLoS One. 2025 May 13;20(5):e0323200. doi: 10.1371/journal.pone.0323200 (PMC12074262; doi:10.1371/journal.pone.0323200)
Supplement: S3 Table — (DOCX) [file pone.0323200.s005.docx]

***Supplementary Table 3****. Yearly numbers of patients in intensive care due to COVID-19 and with COVID-19 and the number and proportion of unvaccinated patients*

|  | No patients due to COVID-19 | No (%) of patients unvaccinated | With COVID-19 | No (%) of cases unvaccinated | Total |
| --- | --- | --- | --- | --- | --- |
| 2020 | 22 | 22 (100%) | - | - | 22 |
| 2021 | 102 | 90 (88%) | 3 | 2 (67%) | 105 |
| 2022 | 58 | 34 (58%) | 57 | 8 (14%) | 115 |
| Total | 182 | 146 (80%) | 60 | 10 (17%) | 242 |
