# Supplementary material for: Economic impact and disease burden of COVID-19 in a tertiary care hospital: A three-year analysis
Source: PLoS One. 2025 May 13;20(5):e0323200. doi: 10.1371/journal.pone.0323200 (PMC12074262; doi:10.1371/journal.pone.0323200)
Supplement: S4 Table — (DOCX) [file pone.0323200.s006.docx]

***Supplementary Table 4****. Total number of hospital care days on TUH during the comparator years 2018-2019 and during the 2020-2022 COVID-19 pandemic*

|  | 2018 | 2019 | 2020 | 2021 | 2022 |
| --- | --- | --- | --- | --- | --- |
| No of days | 270 404 | 266 454 | 247 289 | 257 425 | 244 389 |
