# Supplementary material for: Economic impact and disease burden of COVID-19 in a tertiary care hospital: A three-year analysis
Source: PLoS One. 2025 May 13;20(5):e0323200. doi: 10.1371/journal.pone.0323200 (PMC12074262; doi:10.1371/journal.pone.0323200)
Supplement: Attachment — Submitted filename: Economic Impact and Disease Burden of COVID.docx [file pone.0323200.s007.docx]

Economic Impact and Disease Burden of COVID-19 in a Tertiary Care Hospital: A Three-Year Analysis

**Summary comments:** This is a very relevant paper and promises to be an important contribution to the literature. I believe that there a few methodological issues and some areas that can be strengthened in the discussion to show the relevance of the paper and the important policy contribution it makes. With these proposed changes made I would recommend that the publisher considers it for publication after revisions have been made.

# Detailed comments

**Relevance:** This paper is a very important contribution to the literature on the economic burden of COVID 19 as it will inform planning for future outbreaks mitigation measures. The presentation of the economic burden of COVID 19 cases is critical for hospitals to prepare adequately for future scenarios.

**Methodology:** The following was noted about the methodology of the study:

The data sources were well spelt out as well as the data that was used for the costing study. The sources are credible and verifiable. The time horizon of the study was well spelt out. The Identification of costs was well done for many of the cases under consideration including (hospitalization for COVID 19 patients. Furthermore, the inclusion of the opportunity costs related to extra inputs for management of COVID 19, as well as the costs of foregone care for elective surgery is good. The authors should consider addressing the following:

- It is also important to highlight the perspective that is driving the costing study. By including indirect costs, one would assume that some patient related costs would be included but these are not included. The scope of the costs covered and the justification for the choice of perspective should be included in the study.
- There was no mention of whether costs were discounted and what rate of discounting was used. In the event that authors took a deliberate choice not to discount, there is need for the authors to justify why.
- The presentation of the economic burden of COVID 19 cases is critical for hospitals to prepare adequately for future scenarios. However, COVID 19 varied in severity and the costs associated with each severity profile varied markedly, it would be important to stratify the costs by severity profile.

It is also important to highlight whether these include costs of long-term effects of COVID 19. I suggest that the description of what a COVID 19 patient is made clearer highlighting the eligibility and inclusion criteria for who was considered a COVID 19 case.

- Kindly indicate medication costs for those who came in ED and those who were hospitalized for other reasons and then got COVID 19.

Discussion section:

- Lines 274-280: Kindly present equivalent costs for the currencies that are non-Euro costs. Furthermore, the discussion should be presented better. The costs are similar to the hospital in Spain and somewhat similar to that in USA. These countries are of similar income profile to Finland. While those with lower costs Mexico and Brazil are in a different income bracket. Could this be an explanation? Kindly discuss a bit further,
- Lines 281-285: How do these findings compare to findings from other hospitals in Finland, Europe or other continents? Do they confirm what you have found? If not, why not?
- Lines 281-309: This section is restating what has been described in the results section. The authors should discuss how this compares with findings from Finland or other settings and what the implications of their findings are and how they contribute to the evidence-base for hospital and government efforts on pandemic preparedness and response going forward.
- Kindly also indicate some future research areas that your research highlights.
