# Supplementary material for: Economic impact and disease burden of COVID-19 in a tertiary care hospital: A three-year analysis
Source: PLoS One. 2025 May 13;20(5):e0323200. doi: 10.1371/journal.pone.0323200 (PMC12074262; doi:10.1371/journal.pone.0323200)
Supplement: Attachment — Submitted filename: Review _comments .docx [file pone.0323200.s008.docx]

**Comments**

1. There were WASH activities as prevention of infection in most health facilities, while primarily focused for COVID-19 pandemic, they have great value in preventing other infections. At the same time, what are the expected gains that has to be deducted from the costs?
2. Although the report mentions lost income due to cancelled elective surgeries, it could benefit from a more thorough analysis of the long-term implications of these cancellations on patient care and hospital finances.
3. The findings would be strengthened by more rigorous statistical analyses to assess the significance of observed trends and differences between groups.
4. While the study briefly mentions limitations, a more comprehensive discussion of potential confounding factors and the generalizability of the results would enhance the credibility of the findings.
5. The exclusion of outpatient SARS-CoV-2 NAAT costs and communal primary care costs limits the study's scope, underestimating the total economic impact of the pandemic on the healthcare system.
